# Dietary Practice among the Patients with End Stage Renal Disease undergoing Maintenance Haemodialysis

**DOI:** 10.31729/jnma.3723

**Published:** 2018-10-31

**Authors:** Sangita Lamichhane (Dulal), Manasa Thapa Thakurathi, Ram Krishna Dulal, Shreejana Karki, Krishna Bahadur Raut

**Affiliations:** 1 National Kidney Center, Kathmandu, Nepal; 2 Nepal Padma Kanya Multiple Campus, Bagbazar, Kathmandu, Nepal; 3 National Open College, Pokhara University, Nepal; 4 Department of Pneumology, Lungenklinik Hemer, Hemer, Germany; 5 Department of Emergency, Chitwan Medical College, Chitwan, Nepal

**Keywords:** *dietary counselling*, *ESRD*, *haemodialysis*, *kidney*, *plant-based diet*, *nutrition*

## Abstract

**Introduction:**

Several studies on the nutritional status of chronic kidney disease patients living on haemodialysis revealed high prevalence of malnutrition (18–94%). A hospital-based study in Nepal revealed 66.7% mild to moderately malnourished and National Kidney Center reported common protein-energy malnutrition problem among haemodialysis patients. As almost all patients undergoing maintenance haemodialysis looked malnourished, this study was carried out to explore prevailing dietary knowledge and practice of the patients.

**Methods:**

A descriptive cross-sectional survey of 471 patients undergoing haemodialysis from June 2015 to July in 2015 was carried out. After stratification through a systematic random sampling method, 40 (67%) patients from National Kidney Center and 20 (33%) from Bir Hospital were selected, interviewed face to face by using structured questionnaire. Ethical and institutional approval and patients' consent were obtained.

**Results:**

The level of knowledge score found to be medium and practice score was even low. Seventy percent knew about renal diet but only 36 (60%) believed in it. After having kidney disease 42 (70%) had changed their dietary practice. Surprisingly, 38 (63.3%) said they ate the food what the other member in their family ate.

**Conclusions:**

Considerable limited knowledge (medium) and practices (low) scores were found. Had they have taken adequate dietician's support, they might have taken right kind and right amounts of foods and benefitted in controlling potassium, phosphate and protein at the recommended level. Plant-based diet could assist in end stage renal disease in a number of ways: an edge of protection against diet cost, reduce inter-related co-morbidities or complications (hypertension and diabetes).

## INTRODUCTION

A global Chronic Kidney Disease (CKD) prevalence was estimated to be 8–16% and affecting 8–16% adult population in Asia.^1, 2^ CKD was detected in 10.6% in eastern Nepal and the prevalence of ESRD was estimated to be 2600 cases/year in Nepal.^3, 4^

CKD has strongest death risk factor compared to other condition due to protein-energy wasting, including a relatively low serum albumin and fat or muscle wasting.^5^ A poor pre-dialysis nutritional status increases morbidity and mortality after initiation of renal replacement therapy.^6^ Studies on CKD revealed evidence of malnutrition/wasting problem in 18–75%.^7–8^ The signs of malnutrition reported in 23–76% in haemodialysis and 1850% in peritoneal dialysis patients.^9, 12^

A study in Nepal revealed 66.7% mild to moderately malnourished and National Kidney Center (NKC) reported a common protein-energy malnutrition among haemodialysis patients.^13, 14^ Though the nutrition is regarded as an essential element of the dialysis prescription-^15^ evaluation of the nutritional status of dialysis patients is not considered as an important part of day-to-day practice.^16^

Since NKC considers nutritional status as an important factor for ESRD- this study attempted to explore the dietary knowledge and practice of those patients living on haemodialysis.

## METHODS

A descriptive cross-sectional survey on ESRD patients undergoing haemodialysis treatment at National Kidney Center and National Academy of Medical Sciences (NAMS) thereafter referred as Bir Hospital- Kathmandu, Nepal was carried out during the month of June 2015 and July 2015. Ethical approval was obtained from concerned Research Committee of National Kidney Center of Health Care Foundation-Nepal and National Academy of Medical Sciences (NAMS)- Bir Hospital authority.

During the study- a total of 60 patients at Bir Hospital and 411 patients at National Kidney Center were undergoing haemodialysis treatment. With a margin of error of 0.10, a confidence level of 90% and a population proportion of 0.5 (since the prevalence of knowledge and practice in ESRD patients was unknown)-we calculated a sample size of 60. The systematic sampling method was adopted after stratification (Bir Hospital = 33% and National Kidney Center = 67%), in which individual patient was selected for interview and then the subsequent patient was chosen applying a systematic interval until the desired sample size was reached.^17–18^


$$\begin{array}{l} \text{Sample size (n):}{\text{z}}^{2}\times {\text{pq/e}}^{2}\\ =60 \end{array}$$

Where z: Value of z at confidence interval of 90%, z = 1.645
p = 50% prevalence
q = 1-p
e = margin of error- 10%

Since it was a new study in the context of Nepal- the questionnaires were generated by reviewing similar studies conducted in other countries and also reviewing various relevant articles on ESRD and diet. Then the content experts in the field of nutrition science reviewed the questions and objectives before using it. The study was undertaken after doing a pilot test of questionnaires in 20 patients undergoing haemodialysis treatment at National Kidney Center. The reliability test of the tool was done, where Cronbach Alpha Coefficient was (r = 0.75). It showed that repeatability was good indicating that these tools would produce stable and consistent results on knowledge on ESRD- renal diet-attitude towards it and dietary practice. Face validity appeared to measure what it intended to measure.

Subsequently- the purpose of the research was explained to each participant individually prior to the interview. All were assured of privacy and confidentiality. Those who could read and write gave written consent and verbal consent was obtained from those who could not read and write.

Since high-quality cross-sectional studies are susceptible to bias- we considered controlling selection bias by considering eligibility (ESRD patients aged 18 years and above- both sexes male and female- consented to participate willingly- who had at least 15th session dialysis already and Nepali citizen and receiving dialysis twice per week). Then- applied simple random sampling after stratification so that every patient, at both the strata (Bir Hospital and National Kidney Center)- had an equal chance of being included. In order to control information bias- we did not take minor (<108 years)-those unwilling to participate in this study- did not speak and understand the Nepali language- who were getting psychiatric treatment additionally and known case of depression in medical record file.

At first, obtained data was entered in to the Microsoft Excel 2007 and analysed by using Statistical Package for the Social Sciences- version 18.0. An association between knowledge and practice with their selected demographic variables are presented in frequency distribution and percentages.

## RESULTS

The demographic characteristics of ESRD patients undergoing haemodialysis treatment at National Kidney Center and Bir Hospital studied were age- sex- ethnicity-education level and marital status- where 34 (56.7%) revealed aged 50 years and above- 40 (66.67%) were male and only 20 (33.33%) were female.

About 45 (75%) of the respondents said they knew about renal diet. Among the respondents- 24 (40%) said they did not believe in renal diet. About 41 (69.3%) said meat was not good for the ESRD patient and 44 (73.3%) said “red meat is harmful for ESRD” case. Forty-seven (78.3%) said drinking excess milk was not good for chronic kidney disease. Among the respondents, only 30 (50%) of them said that they knew which particular fruits could be consumed daily. Similarly, about 28 (47.7%) said they did not know spinach was harmful for ESRD patients living on haemodialysis. More than a quarter of subjects 16 (26.7%) did not know the need of salt intake reduction. Only 31 (51.7%) of them knew that they could enjoy food during haemodialysis. Thirty-six (60%) of the ESRD patients knew and visited the dietician for a dietary counselling (Table 1).

**Table 1.** Self-rated knowledge level and diet-related dimensions.

S. No.	Particulars	Yes n (%)	No n (%)
1.	Do you know about renal diet?	45 (75)	15 (25)
2.	Do you believe that renal diet is useful for ESRD patient?	36 (60)	24 (40)
3.	Do you think meat is good food for chronic kidney disease?	19 (31.7)	41 (69.3)
4.	Do you know red the meat is harmful for ESRD?	44 (73.3)	16 (26.7)
5.	Do you think milk intake in excess amount is good for CKD?	13 (21.7)	47 (78.3)
6.	Do you know which fruits you can be eaten daily by ESRD?	30 (50)	30 (50)
7.	Do you know that spinach is harmful for ESRD living on haemodialysis?	32 (53.3)	28 (47.7)
8.	Do you know why salt intake is to be limited in chronic kidney disease?	44 (73.3)	16 (26.7)
9.	Do you know commercially packed food item is harmful to chronic kidney patient?	42 (70)	18 (30)
10.	Do you know roasted and salted nuts and seed are harmful to ESRD (as gives more sodium)?	47 (78.3)	13 (21.7)
11.	Do you know about need of controlling fluid intake in ESRD (even CKD) case?	55 (91.7)	5 (8.3)
12.	Do you think during dialysis everything can be eaten?	31 (51.7)	29 (49.3)
13.	Did you visit the Dietician for dietary counselling (if you know about Dietician)?	36 (60)	24 (40)

This study resulted that 36 (60%) of the patients had no knowledge about the potassium containing food items and 47 (78.3%) did not have idea about controlling the level of potassium in blood (Table 2). Similarly, 39 (65%) did not know about phosphorus-containing food & itching caused by elevated phosphorus. Almost 52 (86.7%) did not know about darkling (darkness) of skin when phosphorus level gets increased in the blood. Only 19 (31.7%) of them said they knew that refined flour was not prescribed for renal patients.

**Table 2.** Knowledge about minerals sources and its control.

S.No.	Particulars	Yes n (%)	No n (%)
1.	Do you know about the potassium sources food items	23 (38.3)	36 (60)
2.	Do you have idea how to control level of potassium in blood	13 (21.7)	47 (78.3)
3.	Do you know phosphorus-containing food causes itching in kidney patient	21 (35)	39 (65)
4.	Do you know high phosphorous-containing food causes darkling (darkness) of skin to kidney disease	8 (13.3)	52 (86.7)
5.	Do you know refined flour is not prescribed for renal patient	19 (31.7)	41 (68.3)

Among the respondents, 42 (70%) said they changed dietary practice after having kidney disease (Table 3). Notably, 38 (63%) of the ESRD patients said they ate the same food as other member in their family. Twelve (20%) consumed meat, 5 (8.3%) drank more than two cups of milk in a day, 16 (26.7%) consumed commercial food item, 14 (23.3%) consumed orange daily. About 8 (13.3%) said they added extra salt in their meal, 17 (28.3%) consumed regularly food made from refined flour, 54 (90%) consumed low salt food, 12 (20%) consumed junk food (ready-made or ready to eat food) from the market, 9 (15 %) drank water more than three litres in a day. Among the respondents, only 18 (30%) said they applied potassium-leaching process.

**Table 3.** Dietary practice of ESRD.

S. No	Particulars	Yes n (%)	No n (%)
1.	Do you consume meat every day	12 (20)	48 (80)
2.	Do you drink milk more than two cups in a day	5 (8.3)	55 (91.7)
3.	Do you consume commercial food item in daily snack	16 (26.7)	44 (73.3)
4.	Do you consume orange regularly	14 (23.3)	46 (76.7)
5.	Do you consume regularly food made from refined flour	17 (28.3)	43 (71.7)
6.	Do you consume extra salt in meal	8 (13.3)	52 (86.7)
7.	Do you control diet for your renal disease	44 (73.3)	16 (26.7)
8.	Do you consume low salt food	54 (90)	6 (10)
9.	Do you consume junk food (ready-made packed) food from market	12 (20)	48 (80)
10.	Do you drink water more than three litres a day	9 (15)	51 (85)
11.	Have you changed dietary practice after having kidney disease	42 (70)	17 (28.3)
12.	Do you consume same food what the other member in your family eat?	38 (63.3)	22 (36.7)
13.	Do you practice potassium leaching process	18 (30)	42 (70)

It is evident that about 11 (68%) respondents considered that the meat was not good for kidney disease, whereas 12 (20%) told they consumed meat every day, 23 (38.3%) said they knew about the source of potassium food item, but 34 (56.7%) consumed potatoes and yam in excess amount. Only 13 (21.7%) knew about controlling the potassium level in blood and 42 (70%) of them did not apply potassium leaching process in their daily life (Table 4). Forty-four (73.3%) said they knew about cause of salt reduction in kidney disease, whereas 8 (13.3%) consumed extra salt in their meal. Of them, 55 (91.7%) knew about controlling fluid intake in CKD, whereas 9 (15%) drank more than three litres of water in a day.

**Table 4.** Knowledge and practice relationship.

S. No	Knowledge	Yes n (%)	No n (%)	Practice	Yes n (%)	No n (%)
1.	Do you think meat is good food for chronic kidney disease?	19 (31.7)	41 (68.3)	1. Do you eat meat every day	12 (20)	48 (80)
2.	Do you know about the highest potassium sources from food items?	23 (38.3)	36 (60)	2. Do you eat potatoes and yam excess	34 (56.7)	26 (43.3)
3.	Do you know about controlling potassium level in blood	13 (21.7)	47 (78.3)	3. Do you practice potassium leaching process	18 (30)	42 (70)
4.	Do you know the reason of salt intake reduction in kidney disease	44 (73.3)	16 (26.7)	4. Do you consume extra salt in meal	8 (13.3)	52 (86.7)
5.	Do you know about controlling fluid intake in CKD case	55 (91.7)	5 (8.3)	5. Do you drink water more than three litres a day	9 (15)	51 (85)

## DISCUSSION

The government of Nepal has nutritional program running under the umbrella of Ministry of Health and Population. But disease-specific guidelines for non-communicable diseases like diabetes, hypertension and CKD are not available as yet. Balancing the nutritional status among CKD patients is very vital to those undergoing haemodialysis treatment. Generally, as haemodialysis patients have reduced appetite, their attending physician or nephrologists are the first point to get nutritional advice.

When kidneys can't do their function themselves, human body can't remove extra fluids and waste products from bloodstream, restore electrolyte levels, and help in controlling blood pressure. Dialysis does some of the work of the kidneys by filtering harmful wastes, salts and fluid from the blood.^19^ Generally, in early stage of chronic kidney disease, there may not be any signs or symptoms. As chronic kidney disease progresses to end-stage renal disease, signs and symptoms might appear.^20^ Then, the one and only immediate solution is to put the patients on dialysis.

The chronic diseases - heart disease, diabetes, some kinds of cancer, dental disease, and adult bone loss, all have a connection to poor diet. These diseases cannot be prevented by a good diet alone; they are to some extent determined by a person's genetic constitution, activities, and lifestyle. Within the range set by individual's genetic inheritance, however, the likelihood of developing these diseases is strongly influenced by persons' daily choices.^20^

The ESRD patients' energy requirement vary greatly, depending on the underlying cause of the renal failure and any other comorbidity. As nutritional status is an important predictor of outcome in ESRD patients living on maintenance HD-^21^ this study aimed at determining the dietary knowledge and practice among those patients living on haemodialysis treatment at National Kidney Center and National Academy of Medical Sciences- Bir Hospital (NAMS- Bir Hospital)- Kathmandu- Nepal.

Though the nutritional status of individuals depends on multi-factors e.g. food accessibility- affordability-nutrition knowledge- the nutritional education-adherence of education and practice can make a huge difference.

In this study about 43% respondents aged 18 to 50 years were living on HD-which is a highly productive age group. Aged 50 and above revealed 57% with the ESRD. But in an earlier study conducted in Nepal reported that aged 41–60 constituted 45.8%- where the majority respondents were male (64.6%).^22^ A study done on at Nepal Medical College and Teaching Hospital-Kathmandu revealed that 57% were male. Ghimire M et al. studied 50 ESRD patients- in which they found the age ranging from 18–79 years and males 64%.^23^ The majority of the respondents were male (66.67%) in our study- which indicates that male are still ahead of female in seeking treatment. Both the male and female patients can live on maintenance haemodialysis and contribute immensely in the nation-building activities given that they are well and balanced-nourished.

Another largest retrospective cohort conducted in Nepal (1991–2000) found that the mean age of patients were 46.7±16.7 years with age ranging from 8 to 89 years. Of which- 905 (65%) patients were male. Similar to other studies in India (67.8% male and 32.2% female)-Saudi Arabia (64.5% male and 35.5% female) and Japan (54.4% male and 45.6% female).^24^ This trend seem to be quite relevant to our study- where higher age group male are found compared to women. According to Centers for Disease Control and Prevention- the risk of CKD also increases with an increasing age and men with CKD are 50% more likely than women to have kidney failure.^25^

The patients said they knew and also felt about the pre-symptoms before their ESRD was diagnosed. About 46% said that swelling was seen on legs-eyelids, face flowed by nausea, vomiting 13.3%, loss of appetite- headache and Insomnia 10%- back pain 3.3% and weight loss 1.7%. In fact- a person can lose a lot of kidney function before symptoms of kidney disease occur.^26^ Ghimirey A et al. found that stage V CKD (ESRD) patients having co-morbid conditions of hypertension in 79.6% and diabetes mellitus in 15.6% and in 4.7% both hypertension with diabetes mellitus. But none of the patients were on medications for the management of their disease states.^27^

It is reported that diet-related decisions are influenced by multiple factors, including taste, financial constraints, individual preferences- social status- education level-societal norms- health- and relationships- trust in food sources- and convenience. Dietary preferences and behaviours are highly individual.^28–32^ The scenario of our context might not be exception to it. In addition to the above- it could be due to lack of awareness of patients on renal diet and the involvement of the clinical dietician in managing ESRD care. Since this study was the cross-sectional pattern- it does not allow for the establishment of cause and effect relationships. Therefore- future studies aiming at exploring casual relation would be of helpful.

In this study- majority of patients 91.7% said that less water intake would help in managing kidney disease. Patients should limit fluid 1 to 1.5 litres a day and water intake to avoid fluid overload in the body. Personalized fluid consumption goals and education can improve the patient compliance with dietary restrictions.^33^

Although studies are small- several support the idea that plant-based diets can delay the progression of CKD- provide endothelial protection- control high blood pressure- and decrease proteinuria. These days-our dialysis patients seldom die secondary to high potassium or uremia.^26^

Researchers recommended to promote plant-based diet for ESRD as well as all types CKD patients- which can provide nutrients that not only assists in ESRD but also can provide an edge of protection against diet cost-reduce inter-related co-morbidities or complications (diabetes and hypertension).^34^

A consulting clinical dietician at Gig HarbourWashington- USA reported although the studies are small in size- several evidence support the idea that plant-based diets can delay even the progression of CKD- provide endothelial protection- control high blood pressure- and decrease proteinuria. These days- dialysis patients seldom die secondary to high potassium or uremia.^35^

Michael Greger reported that the researcher at Harvard University followed thousands of women- their diet and their kidney for more than a decade that concluded animal protein was the culprit in declining kidney function. Studies reported that kidney appeared to handle plant protein very differently from animal protein and plant protein was found to help preserve function in ailing kidneys. Animal protein tends to have a higher level of sulfur-containing amino (methionine)- which produces sulfuric acid when metabolized in the body. Worst offenders: fish (including tuna), pork, poultry, cheese. An equivalent amount of plant protein causes no noticeable stress on kidneys. In contrary- fruit and vegetables are generally base forming- which helps neutralise acids in our kidneys and eating normal whole foods can return dopamine sensitivity to normal levels.^36^ It is still a “Thorny” issue since the influence of most dietary interventions has been examined in small studies in patients with CKD. A careful renal diet management could be considered until a plant-based diet will be examined through large randomized controlled trials in patients with CKD.

## CONCLUSIONS

An effective strategy to enhance the dietary management is inevitable by adhering to dietary restriction but through a proper diet therapy without causing malnutrition means assuring the daily calorie-vitamin- minerals and protein in-take- adequate energy.

Thus- it is vitally important for the ESRD patients to know about the importance of dietician's role since they could benefit tremendously by visiting dietician for a proper dietary counselling and nutritional management.
